# Psychometric Validation of the Spanish OSAKA Questionnaire: A Cross-Sectional Study Among Colombian Dental Professionals

**DOI:** 10.3390/dj13070329

**Published:** 2025-07-20

**Authors:** Olga Patricia López-Soto, Juan Alberto Aristizabal-Hoyos, Jackeline Mulett-Vásquez, Héctor Fuentes-Barría, Raúl Aguilera-Eguía, Lissé Angarita-Davila, Diana Rojas-Gómez, Ángel Roco-Videla

**Affiliations:** 1 Departamento de Salud Oral, Facultad de Salud, Universidad Autónoma de Manizales, Caldas 170008, Colombia; sonrie@autonoma.edu.co (O.P.L.-S.); jaristi@autonoma.edu.co (J.A.A.-H.); jackymulett@autonoma.edu.co (J.M.-V.); 2 Vicerrectoría de Investigación e Innovación, Universidad Arturo Prat, Iquique 1110939, Chile; 3 Escuela de Odontología, Facultad de Odontología, Universidad Andres Bello, Concepción 3349001, Chile; 4 Departamento de Salud Pública, Facultad de Medicina, Universidad Católica de la Santísima Concepción, Concepción 3349001, Chile; raguilerae@ucsc.cl; 5 Escuela de Nutrición y Dietética, Facultad de Medicina, Universidad Andres Bello, Concepción 3349001, Chile; lisse.angarita@unab.cl; 6 Escuela de Nutrición y Dietética, Facultad de Medicina, Universidad Andres Bello, Santiago 7550000, Chile; diana.rojas@unab.cl; 7 Dirección de Desarrollo y Postgrados, Universidad Autónoma de Chile, Galvarino Gallardo 1983, Santiago 7500138, Chile; angel.roco@uautonoma.cl

**Keywords:** sleep apnea obstructive, surveys and questionnaires, health knowledge, attitudes, practice, psychometrics, dentists

## Abstract

**Objectives**: To evaluate the psychometric properties of the Spanish version of the obstructive sleep apnea knowledge and attitudes (OSAKA) questionnaire among dental professionals in Colombia. **Methods:** A cross-sectional study was conducted in Manizales, Colombia, between January and June 2024. A total of 120 dental professionals, including general dentists, specialists, residents, and academic clinicians, were selected through stratified random sampling. Participants completed the Spanish-adapted OSAKA questionnaire and a structured sociodemographic survey. Internal consistency was assessed using Cronbach’s alpha (α) and McDonald’s Omega (Ω). Construct validity was examined through exploratory factor analysis (EFA), with Kaiser Meyer Olkin (KMO) values and Bartlett’s test. **Results:** The knowledge subscale (18 items) showed good reliability (α = 0.83; Ω = 0.83), while the attitude subscale (5 items) showed near-acceptable reliability (α = 0.68; Ω = 0.59). KMO values were 0.79 for knowledge and 0.57 for attitudes; Bartlett’s test was significant in both cases (*p* ≤ 0.001). EFA supported structural validity: six components with eigenvalues >1 explained 61.66% of the variance for knowledge, and two components explained 79.49% for attitudes. Significant differences in attitude scores were found among professional groups (*p* ≤ 0.001, η^2^ = 0.13), with specialists scoring highest ($\bar{X}$ = 17.66) and general dentists lowest ($\bar{X}$ = 14.62). Knowledge scores did not significantly differ between groups (*p* = 0.47). **Conclusions:** The Spanish OSAKA questionnaire is a valid and reliable tool to assess knowledge and attitudes toward obstructive sleep apnea in Colombian dental professionals. Its use may support improved education, screening, and interdisciplinary collaboration in dental settings.

## 1. Introduction

Obstructive sleep apnea (OSA) is a prevalent respiratory disorder characterized by repeated episodes of partial or complete upper airway obstruction during sleep. This condition leads to intermittent hypoxemia, sleep fragmentation, and alterations in sleep architecture [1] and is associated with multiple cardiovascular, metabolic, and neuropsychological consequences that significantly impair quality of life and increase global socioeconomic costs [2,3]. Indeed, undiagnosed or untreated OSA has been linked to cardiovascular disease, mental health disorders, and traffic accidents associated with excessive daytime sleepiness, making it a critical public health issue [4,5,6]. The combined prevalence of OSA is estimated at approximately 54%, with a marked increase with age [7].

Although polysomnography remains the gold standard for diagnosing OSA [8,9], its routine application is often hindered by logistical, economic, and patient tolerance barriers. These limitations have prompted the development of alternative diagnostic methods such as home sleep apnea screening devices (HSAT) [10,11], among which clinical screening questionnaires have gained recognition as practical and accessible tools for the presumptive identification of individuals at high risk for OSA [12,13,14,15]. Given their frequent and prolonged contact with patients, dental professionals are in a strategic position to contribute to the early diagnosis and interdisciplinary management of this condition [16,17]. Through the examination of oral and oropharyngeal anatomical structures—such as the tongue, uvula, tonsils, and palatal shape—during routine clinical assessments, dentists can identify signs suggestive of OSA risk [18]. Moreover, they may administer standardized screening tools or implement specific questionnaires, facilitating timely referral to sleep medicine specialists [19]. Dentists also play an active role in treatment by fabricating and monitoring mandibular advancement devices in coordination with other healthcare professionals [20]. However, for these actions to be effective, solid knowledge of the disease and a positive attitude toward its management are essential.

Despite its clinical relevance, the level of knowledge and attitude of dental professionals regarding OSA has been insufficiently explored [17,21,22]. Most recent studies have focused on medical professionals, with only a limited number specifically addressing the dental population [21,22]. Although dentists generally demonstrate a favorable attitude toward OSA, there remains notable heterogeneity in their knowledge regarding its diagnosis and treatment [23]. Additionally, their willingness to actively participate in managing the condition is often constrained by limited clinical experience in the field [16]. This situation is particularly relevant in the Latin American context, where OSA is emerging as a growing public health concern—especially in countries like Colombia, with significant implications for population quality of life and overall well-being [24]. In this scenario, the availability of valid and reliable instruments to assess the knowledge and attitudes of oral health professionals toward this condition becomes a priority.

One of the most widely used tools for this purpose is the obstructive sleep apnea knowledge and attitudes (OSAKA) questionnaire, whose Spanish version has been previously validated in countries such as Peru, Venezuela, and Ecuador [25]. However, its applicability and validity in the Colombian dental context had not yet been explored. In response to this gap, the present study aimed to analyze the psychometric properties of the OSAKA questionnaire in a sample of oral health professionals in Colombia to determine its validity, reliability, and suitability as an evaluative instrument in clinical and academic settings within the country.

## 2. Materials and Methods

### 2.1. Design

A cross-sectional observational study with a quantitative and methodological scope was conducted to evaluate the structural validity and internal consistency of the Spanish version of the OSAKA questionnaire [25]. This study was designed following the STROBE guidelines for observational studies [26]. The protocol was approved by the Ethics Committee of the Autonomous University of Manizales (Protocol code: GIN-FOR-003; Approval date: 22 March 2023) and complied with the Colombian Ministry of Health Resolution No. 8430 of 1993 as well as the ethical principles of the Declaration of Helsinki [27].

### 2.2. Context

In Colombia, OSA and other sleep-related disorders represent a growing public health issue with significant impacts on quality of life and overall well-being [24]. Despite their clinical relevance, the availability of validated instruments in Spanish or Portuguese for their assessment in Latin America remains limited [16,25]. In response, several transcultural adaptations of the OSAKA questionnaire have been developed and validated in Spanish, recognizing its utility in supporting research, professional training, and the quality of clinical care [25]. However, the applicability and validity of the OSAKA instrument in specific contexts, such as among residents, general practitioners, and dental specialists in Colombia, still required exploration, especially considering that academic training and clinical practices may differ substantially from the populations in which the original versions were validated.

### 2.3. Participants

The study was conducted between January and June 2024 in ambulatory dental clinics across private, governmental, and academic sectors located in the urban area of Manizales, Colombia. Active clinical dental professionals were included and classified into the following four categories: university clinical faculty, residents in specialization programs, specialists, and general dentists.

A total of 120 participants were selected through stratified random sampling to ensure proportional representation of the different professional categories in the population.

It is worth noting that participation was conducted using a mixed-format approach, through either a face-to-face interview carried out by the researchers or an online form hosted on the Google Forms platform. In both modalities, written informed consent was obtained. Importantly, no participants were lost during the follow-up process, regardless of the participation method; therefore, the final sample included the same 120 individuals initially selected. The inclusion criteria were as follows: currently active clinical dentists, residents, and faculty members with clinical duties in Manizales. Exclusion criteria included inactive professionals, undergraduate students, and incomplete or duplicate survey responses.

All participants were informed about the study objectives, inclusion criteria, and the confidentiality of their data and provided written informed consent.

### 2.4. Variables, Instrument, and Data Sources

The primary instrument was the OSAKA questionnaire, previously adapted and validated to assess health professionals’ knowledge and attitudes regarding the identification and management of patients with obstructive sleep apnea [22,28]. The instrument has been widely used in various clinical and academic contexts, and its Spanish version has been validated in several Latin American countries [21,25,29]. It comprises 23 items divided into the following two sections.

Knowledge: These items cover fundamental domains such as epidemiology, pathophysiology, clinical manifestations, diagnostic methods, and therapeutic options. Response options include “true”, “false”, or “I don’t know.” Each correct answer is awarded one point. Notably, the correct response to items 3, 4, 5, 6, 7, 9, 10, 11, 13, 14, 16, 17, and 18 is “true”; incorrect answers and the “I don’t know” option (included to reduce guessing bias) do not contribute to the score. In total, knowledge scores range from 0 to 18 points [25].

Attitude: This section includes 5 items assessed using a 5-point Likert scale, where 1 indicates “strongly disagree” and 5 “strongly agree”. The total attitude score is calculated by summing the individual item scores, resulting in a possible range of 5 to 25 points. Higher scores indicate a more positive and favorable attitude toward the diagnosis and management of OSA [25].

In addition, a structured form was used to collect the following sociodemographic variables: sex, age, professional category, and years of clinical experience. All data were collected anonymously using Google Forms platform.

### 2.5. Sociodemographic Variables

These included the following: sex (male/female), age (years), professional category (general dentist, specialist, resident, clinical faculty), and years of clinical experience. This information enabled contextualization of findings, identification of differences across professional subgroups, and exploration of associations with knowledge and attitude scores toward OSA.

### 2.6. Bias

This study presents several limitations and potential sources of bias that should be considered when interpreting the results.

Geographic bias: The study was conducted exclusively in an urban setting, which limits its generalizability to rural areas or regions with different sociodemographic conditions [30].

Uncontrolled variables: Prior formal training in sleep medicine was not assessed, which may act as a confounding variable influencing the results [31].

Despite these limitations, the study provides a relevant preliminary insight into the knowledge and attitudes toward OSA among Colombian dentists and offers a foundation for future, larger-scale investigations.

### 2.7. Sample Size

According to the 2018 National Population and Housing Census [32], the urban population of Manizales is approximately 434.000. With an estimated average density of one dentist per 2500 inhabitants in certain regions of Colombia [33], the target population was projected at 174 active clinical dentists in the city. Based on this estimate, the minimum sample size was calculated for a finite population using a 95% confidence level, 5% margin of error, and an expected proportion of 50% (*p* = 0.5). The minimum required sample was 120 participants, which was fully achieved. This sample is considered adequate for psychometric validation studies in health research, particularly when heterogeneity is ensured through stratified sampling, as recommended by best methodological practices [34,35,36].

### 2.8. Statistical Analysis

Statistical analyses were performed using IBM SPSS Statistics version 27.0 (IBM Corp., Armonk, NY, USA). Both descriptive and inferential procedures were applied to assess the psychometric validity and reliability of the OSAKA questionnaire in a sample of Colombian dentists.

Sociodemographic variables were analyzed using frequencies, percentages, means, and standard deviations. Group comparisons by professional category were conducted using Pearson’s chi-square test (for categorical variables such as sex), supplemented by Cohen’s w coefficient. Continuous variables (e.g., years of experience and questionnaire scores) were analyzed using one-way ANOVA with Tukey HSD post hoc tests. Effect sizes were calculated using partial eta-squared (η^2^) and Cohen’s d. The interpretation of effect sizes followed the conventional thresholds: for η^2^, small (0.01), medium (0.06), and large (0.14); and for Cohen’s d, small (0.10), medium (0.30), and large (0.70) [37,38].

An exploratory factor analysis was also conducted using principal component extraction and Varimax rotation. Sampling adequacy was evaluated using the Kaiser-Meyer-Olkin (KMO) index and Bartlett’s test of sphericity. Adequate criteria included KMO > 0.60, a significant Bartlett’s test (*p* < 0.05), and factor loadings ≥ 0.40 for item retention [39]. Internal consistency of the questionnaire was estimated using Cronbach’s alpha coefficient in conjunction with the McDonald’s Omega coefficient, with thresholds for acceptability defined as ≥0.65 [40,41,42].

A significant level of α = 0.05 and 95% confidence intervals were adopted for all analyses. Effect size interpretations followed Cohen’s conventions for the d, η^2^, and w coefficients as previously described [38].

## 3. Results

### 3.1. Sociodemographic Characteristics of the Sample

Table 1 summarizes the main sociodemographic characteristics of the sample, categorized by dental specialty. Regarding sex distribution, a higher proportion of women was observed in all groups, particularly among residents (77.33%) and general dentists (61.70%). Although these differences were not statistically significant (*p* = 0.29), the effect size (w = 0.18) suggests a weak association.

In terms of clinical practice years, notable differences were observed among the groups. Academic professionals reported the highest experience (18 ± 10.95 years), followed by specialists (11 ± 7.31), general dentists (10.23 ± 9.66), and residents (5.90 ± 3.31). This difference was statistically significant (*p* ≤ 0.001), with a medium effect size (η^2^ = 0.12), indicating an unequal distribution of professional experience across training levels. These findings provide relevant context for interpreting differences in knowledge and attitudes regarding OSA.

### 3.2. Performance in Knowledge and Attitude Towards OSA

Table 2 presents descriptive and inferential analyses of the average scores for the knowledge and attitude dimensions, according to dental specialty. In the attitude dimension, more marked differences were observed among the groups. Analysis of variance (ANOVA) revealed statistically significant differences (*p* ≤ 0.001), with a moderate effect size (η^2^ = 0.13). Specialists obtained the highest average score ($\bar{X}$ = 17.66), followed by academics ($\bar{X}$ = 16.73), residents ($\bar{X}$ = 16.57), and general dentists ($\bar{X}$ = 14.62).

Tukey’s post hoc comparisons showed statistically significant differences between general dentists and specialists (*p* ≤ 0.001), with a large effect size (d = 0.89). Medium effect sizes were also observed between general dentists and residents (d = 0.64) and between general dentists and academics (d = 0.67), although these differences were not statistically significant. These findings suggest that, beyond overall results, there are relevant differences in attitude levels toward OSA, highlighting the need to strengthen practical and attitudinal training, especially at the generalist level.

Regarding knowledge scores, no statistically significant differences were observed among the groups.

### 3.3. Internal Reliability of the OSAKA Questionnaire

Table 3 presents the internal reliability analysis of the OSAKA questionnaire as applied to this dental population, evaluating two dimensions—knowledge and attitudes toward OSA. The knowledge subscale, composed of 18 items, yielded a mean score of 32.03 (±6.89) and a Cronbach’s alpha of 0.83, and a McDonald’s Omega coefficient of 0.83, indicating good internal consistency. This suggests that the items consistently assess the level of knowledge about OSA.

In contrast, the attitude subscale, composed of 5 items, had a mean score of 16.11 (±3.53) and a Cronbach’s alpha of 0.68 and a McDonald’s Omega coefficient of 0.59, which is considered acceptable for exploratory studies, although slightly below the ideal threshold of 0.65. Together, these results support the reliability of the instrument to evaluate both knowledge and attitudes of participants toward this respiratory condition.

### 3.4. Exploratory Factor Analysis of the OSAKA Questionnaire

Table 4 displays the results of the exploratory factor analysis performed on the two dimensions of the OSAKA questionnaire—knowledge and attitude. For the knowledge subscale, the KMO measure of sampling adequacy was 0.79, indicating good suitability for factor analysis. Six principal components were extracted, explaining 61.66% of the total variance. This suggests a multifactorial structure consistent with the thematic diversity of the items.

In contrast, the attitude subscale presented a moderately low KMO value (0.57). Nevertheless, Bartlett’s test of sphericity was statistically significant, which supported the identification of a potential bifactorial structure. In this case, two components jointly explained 79.49% of the total variance, indicating an acceptable internal structure, although with some limitations in sampling adequacy.

## 4. Discussion

The findings of this study support the structural validity and internal reliability of the Spanish version of the OSAKA questionnaire when applied in Colombian dental settings. The internal consistency of the knowledge subscale was adequate (α = 0.83; Ω = 0.83) and aligns with previous studies conducted in Latin America, where even lower coefficients have been reported (e.g., α = 0.58). This confirms that the instrument maintains its psychometric robustness when applied to various Spanish-speaking healthcare populations [25].

Regarding the attitude subscale, although acceptable reliability coefficients for Cronbach’s alpha and McDonald’s Omega are generally considered to be ≥0.65, our study reported values close to this threshold (α = 0.68; Ω = 0.59). Nonetheless, such values are deemed acceptable in exploratory studies, particularly, in general, populations and when the scale includes a limited number of items. This is expected because short subscales with few and somewhat heterogeneous items often yield lower reliability coefficients. These findings may reflect the inherent variability of attitudinal constructs and highlight the importance of reinforcing this dimension through targeted educational interventions aimed at improving awareness and engagement [40,41,42,43,44].

From a structural perspective, the exploratory factor analysis revealed a coherent organization of the items, with adequate factor loadings and KMO values above 0.70. These results confirm the relevance of the bifactorial model proposed in previous validation studies [45,46]. Thus, the usefulness of the OSAKA questionnaire in the Colombian dental context is supported by both validity and reliability criteria, extending its applicability beyond the medical field and encouraging its integration into educational programs and clinical screening strategies in dentistry.

Furthermore, the OSAKA questionnaire may serve not only as an initial diagnostic tool to assess knowledge and attitudes toward OSA but also as a valuable resource for continuing education and longitudinal monitoring. Its repeated application over time could help track the impact of educational interventions, identify persistent knowledge gaps, and guide the development of targeted training programs tailored to different professional profiles.

In terms of knowledge levels, the overall mean score was 9.52 points, lower than that reported in the original version of the questionnaire [28] and in previous Latin American adaptations [15,25]. Nevertheless, dental residents in training obtained the highest scores, which could be associated with their recent exposure to updated academic content [47]. Despite this, the differences between groups were not statistically significant (*p* = 0.47), suggesting a relatively homogeneous—though insufficient—distribution of basic knowledge. This reinforces the need to include transversal education on OSA in undergraduate dental programs [23].

In contrast, the differences in the attitude subscale were statistically significant (*p* ≤ 0.001), with a medium effect size (η^2^ = 0.13), indicating greater willingness toward OSA diagnosis and management among specialists and academics compared to general dentists [17]. This disparity has already been identified in the literature as a major barrier to early detection of the disorder, since signs and symptoms may go unnoticed, in general, dental care [17]. Previous studies have highlighted that specialists tend to have greater knowledge of OSA, associated with more advanced training, which allows them to recognize symptoms such as night sweats or snoring as clinically relevant indicators [48,49].

Additionally, it has been documented that more years of professional practice do not necessarily correlate with higher knowledge or a more proactive attitude toward OSA. On the contrary, it could reflect a lack of recent updates, which may delay timely clinical management and compromise patient health [17]. This finding is reinforced by the large effect size observed between general dentists and specialists (d = 0.89), underlining the urgent need to incorporate sleep medicine content into undergraduate programs. Consistently, our demographic analysis suggests that accumulated clinical experience does not necessarily translate into greater preparedness regarding OSA. Prior studies have warned that ongoing education plays a more decisive role than years of professional practice [15,17,25].

Although many dentists claim to understand the function and indications of oral appliances for OSA treatment, a passive or negative attitude toward their clinical implementation persists. This situation is related to educational deficiencies, particularly the limited incorporation of dental sleep medicine content in both undergraduate and postgraduate curricula [16,17,50]. In addition to educational barriers, it is important to recognize that psychological and behavioral factors also play a significant role in patient adherence to mandibular advancement device therapy. Elements such as motivation, perception of treatment effectiveness, and comfort levels with the device are increasingly acknowledged as determinants of therapeutic success. These aspects should be considered in future educational strategies aimed at improving both professional attitudes and patient outcomes [51].

Consequently, strengthening continuing education and specialized training emerges as a strategic axis for reducing care gaps in sleep disorders, especially in populations with high prevalence and low diagnostic suspicion [17,52,53].

Beyond the educational strategies previously mentioned, future interventions could explore additional approaches such as clinical simulations, interprofessional education, and the use of digital learning platforms to enhance long-term knowledge retention and clinical application. These tools may improve the detection and management of OSA across different levels of dental care. Moreover, while the OSAKA questionnaire remains a valuable instrument for assessing knowledge and attitudes, alternative screening tools such as the STOP-BANG, Berlin, or NoSAS questionnaires could be considered in future studies, particularly those aiming to integrate clinical risk stratification with educational assessment. The use of complementary instruments may offer a broader perspective on diagnostic readiness and patient care capacity in dental settings [54,55,56].

This study presents several methodological strengths. First, it represents the first psychometric validation of the OSAKA questionnaire in a sample of Colombian clinical dentists, which constitutes a valuable contribution to the standardized assessment of knowledge and attitudes toward OSA in this population. The rigorous application of exploratory factor analyses, along with high internal consistency, supports the robustness of the instrument in this new context. Furthermore, the inclusion of diverse professional profiles—general dentists, specialists, residents, and academics—allowed the identification of significant and clinically relevant differences.

However, certain limitations must be acknowledged. Self-selection bias may have favored the participation of professionals with greater interest or knowledge of OSA, potentially overestimating the actual results in the target population [30]. Additionally, the predominance of participants from urban and academic settings limits the generalizability of the findings to rural areas or regions with less access to specialized training [31]. Lastly, the lack of inclusion of variables such as prior training in sleep medicine or specific clinical experience with OSA patients prevents a more in-depth analysis of other factors associated with knowledge and attitudes toward this condition [32].

## 5. Conclusions

This study validates, for the first time, the OSAKA questionnaire in a sample of Colombian clinical dentists, demonstrating robust psychometric properties and high internal consistency for assessing knowledge and attitudes regarding OSA. The results highlight not only the usefulness of the instrument in clinical and academic settings but also significant disparities in diagnostic attitudes according to the level of professional training.

These findings underscore the importance of integrating sleep medicine content into dental education, particularly at the undergraduate level, as well as developing educational strategies that promote greater involvement of general dentists in the early detection of OSA.

Further research in this area is recommended through multicenter studies that incorporate confirmatory analyses and explore associated clinical variables in order to consolidate the use of the OSAKA questionnaire as both a diagnostic and educational tool in dentistry.

## Figures and Tables

**Table 1:** Characteristics of the sample studied according to dental specialty (n = 120).

Sex	General Dentist	Resident of Specialization	Specialization	Academic Specialist	*p*-Value	Effect Size
Male	18 (38.30%)	8 (26.67%)	16 (50%)	5 (45.45%)	0.29	0.18 (w)
Female	29 (61.70%)	22 (77.33%)	16 (50%)	6 (54.55%)		
Clinical practice (years)	10.23 ± 9.66	5.90 ± 3.31	11 ± 7.31	18 ± 10.95	≤0.001 *	0.12 (η^2^)

%: percentage, *: *p* ≤ 0.001 indicates statistically significant differences χ^2^ test, w: effect size determined by Cramer’s V, η^2^: Effect size determined by partial eta square.

**Table 2:** Average number of correct answers in the total knowledge and attitude dimension according to dental specialty (n = 120).

**Knowledge**	**n (%)**	$\bar{X}$ **± SD**	**95% CI**	***p*-Value**	**Effect Size (η^2^)**
General Dentist	47 (39.17)	8.85 ± 3.29	7.88–9.82	0.47	0.13
Resident	30 (25)	10.10 ± 2.47	9.18–11.02		
Specialization	32 (26.67)	9.87 ± 3.22	8.71–11.04		
Academic	11 (9.16)	9.73 ± 4.86	6.46–12.99		
Total	120 (100)	9.52 ± 3.26	8.93–10.11		
**Attitudes**					
General Dentist	47 (39.17)	14.62 ± 3.02	13.73–15.51	≤0.001 *	0.13
Resident	30 (25)	16.57 ± 3.11	15.40–17.73		
Specialization	32 (26.67)	17.66 ± 3.88	16.26–19.06		
Academic	11 (9.16%)	16.73 ± 3.50	14.38–19.08		
Total	120 (100)	16.11 ± 3.53	15.47–16.75		
**Comparison** **Attitudes**		$\bar{X}$ **_1_**	$\bar{X}$ **_2_**	***p*-Value**	**Effect Size (d)**
General Dentist	Resident	14.62	16.57	0.07	0.64
	Specialization	14.62	17.66	≤0.001 *	0.89
	Academic	14.62	16.73	0.24	0.67
Resident	Specialization	16.57	17.66	0.57	0.30
	Academic	16.57	16.73	0.99	0.05
Specialization	Academic	17.66	16.73	0.86	0.25

%: percentage, $\bar{X}$: mean, SD: standard deviations, *: *p* ≤ 0.001 indicates statistically significant differences Tukey HSD, η^2^: effect size determined by partial eta square, d: effect size determined by Cohen d.

**Table 3:** Reliability analysis for the OSAKA questionnaire in Spanish.

Dimensions	Number of Items	$\bar{X}$ ± SD	Cronbach’s Alpha	McDonald’s Omega
Knowledge	18	32.03 ± 6.89	0.83 *	0.83 *
Attitudes	5	16.11 ± 3.53	0.68 *	0.59

$\bar{X}$: mean, SD: standard deviations, *: good internal consistency determined by Cronbach’s alpha and McDonald’s Omega.

**Table 4:** Exploratory factor analysis results for knowledge and attitudes dimensions.

Dimensions	Kaiser Meyer Olkin	Bartlett’s Test	Component	Eigenvalue	Variance Explained for Components (%)	Cumulative Variance (%)	Reproduced Correlation (r)
**Knowledge**	0.79 *	X^2^ = 524.68 df = 153 *p* ≤ 0.001 **	1	4.83 ***	26.85	26.85	0.58
			2	1.69 ***	9.40	36.25	0.66
			3	1.28 ***	7.11	43.36	0.61
			4	1.18 ***	6.56	49.92	0.62
			5	1.10 ***	6.12	56.04	0.65
			6	1.01 ***	5.62	61.66	0.81
			7	0.93	5.19	66.85	0.47
			8	0.78	4.31	71.16	0.62
			9	0.72	4.02	75.18	0.72
			10	0.69	3.82	79.00	0.62
			11	0.68	3.79	82.79	0.43
			12	0.57	3.14	85.93	0.68
			13	0.55	3.05	88.98	0.66
			14	0.52	2.89	91.87	0.67
			15	0.46	2.53	94.40	0.46
			16	0.39	2.16	96.56	0.52
			17	0.33	1.83	98.39	0.64
			18	0.29	1.61	100	0.70
**Attitudes**	0.57	X^2^ = 258.20 df = 10 *p* ≤ 0.001 **	1	2.21 ***	44.12	44.12	0.90
			2	1.77 ***	35.37	79.49	0.89
			3	0.58	11.64	91.13	0.68
			4	0.26	5.11	96.24	0.85
			5	0.19	3.76	100	0.66

*: Kaiser Meyer Olkin values ≥0.60 indicate acceptable adequacy, **: Bartlett’s test values *p* ≤ 0.001 indicates statistically significant factorable data, ***: Eigenvalue >1 in Kaiser criterion indicates retains component.

## Data Availability

The raw data supporting the conclusions of this article will be made available by the authors on request.
